# Robotic Milling of Electrode Lead Channels During Cochlear Implantation in an *ex-vivo* Model

**DOI:** 10.3389/fsurg.2021.742147

**Published:** 2021-11-11

**Authors:** Jan Hermann, Fabian Mueller, Daniel Schneider, Gabriela O'Toole Bom Braga, Stefan Weber

**Affiliations:** ARTORG Center for Biomedical Engineering Research, Faculty of Medicine, University of Bern, Bern, Switzerland

**Keywords:** robotic cochlear implantation, electrode lead channel, electrode fixation, robotic surgery, image-guidance, patient-specific planning, *ex-vivo* human cephalic study, robotic milling

## Abstract

**Objective:** Robotic cochlear implantation is an emerging surgical technique for patients with sensorineural hearing loss. Access to the middle and inner ear is provided through a small-diameter hole created by a robotic drilling process without a mastoidectomy. Using the same image-guided robotic system, we propose an electrode lead management technique using robotic milling that replaces the standard process of stowing excess electrode lead in the mastoidectomy cavity. Before accessing the middle ear, an electrode channel is milled robotically based on intraoperative planning. The goal is to further standardize cochlear implantation, minimize the risk of iatrogenic intracochlear damage, and to create optimal conditions for a long implant life through protection from external trauma and immobilization in a slight press fit to prevent mechanical fatigue and electrode migrations.

**Methods:** The proposed workflow was executed on 12 *ex-vivo* temporal bones and evaluated for safety and efficacy. For safety, the difference between planned and resulting channels were measured postoperatively in micro-computed tomography, and the length outside the planned safety margin of 1.0 mm was determined. For efficacy, the channel width and depth were measured to assess the press fit immobilization and the protection from external trauma, respectively.

**Results:** All 12 cases were completed with successful electrode fixations after cochlear insertions. The milled channels stayed within the planned safety margins and the probability of their violation was lower than one in 10,000 patients. Maximal deviations in lateral and depth directions of 0.35 and 0.29 mm were measured, respectively. The channels could be milled with a width that immobilized the electrode leads. The average channel depth was 2.20 mm, while the planned channel depth was 2.30 mm. The shallowest channel depth was 1.82 mm, still deep enough to contain the full 1.30 mm diameter of the electrode used for the experiments.

**Conclusion:** This study proposes a robotic electrode lead management and fixation technique and verified its safety and efficacy in an *ex-vivo* study. The method of image-guided robotic bone removal presented here with average errors of 0.2 mm and maximal errors below 0.5 mm could be used for a variety of other otologic surgical procedures.

## Introduction

Cochlear implantation is a neuro-otologic technique used to restore hearing to profoundly deaf patients with sensorineural hearing loss. A microphone and audio processor are worn around the auricle, and a transmission coil is magnetically connected to the implanted receiver-stimulator. This receiver-stimulator is placed in the temporal region underneath the skin in a subperiosteal pocket, while an attached electrode array is inserted into one of the ducts of the cochlea, specifically into the scala tympani. In the manual surgery, the excessive electrode lead is stored and stabilized with various techniques in the mastoidectomy cavity, an access cavity posterior to the auditory canal, to prevent electrode migration and fatigue breaks through micro-movements. On the temporal bone surface, a groove or split-bridge is created from the mastoidectomy cavity to the recessed implant bed or the subperiosteal pocket.

In the emerging robotic technique of cochlear implantation (1, 2), a small-diameter access tunnel is drilled from the surface of the temporal bone directly to the round window of the cochlea following an optimized trajectory. Because no mastoidectomy cavity exists when performing the robotic procedure, the standard process of stowing the excess electrode lead in the mastoidectomy cavity is not possible. Therefore, a purposeful electrode lead management specific to robotic cochlear implantation is needed. In the first clinical studies of robotic cochlear implantation, a channel was manually milled after cochlear insertion. However, milling near the electrode lead poses a risk for damage to the electrode lead directly and indirectly to the delicate intracochlear structures due to the movements caused by the manipulation. Furthermore, milling after insertion risks bone dust and blood contaminating the cochlea, which increases the risk of damage to the organ (3, 4). Hence, it is advisable to mill before electrode insertion. However, prior to insertion, the exact electrode lead length needed and the potential electrode surplus cannot be ascertained by the surgeon, since the electrode lead is not yet inside the cochlea. This can be resolved with an accurate surgical planning on the medical images taken for the robotic procedure.

A dedicated electrode management and fixation system for robotic cochlear implantation can use the patient-specific intervention planning and the high-precision functionality of the robotic platform and extend from the robotic middle and inner ear access. Using the image-guided approach, the robotic electrode management technique can ensure that the electrode can be immobilized against micromovements and resulting mechanical fatigue. Furthermore, the electrode lead can be protected from external trauma by embedding it within the bone over the whole length. We assess that adequately sized margins to vital structures in the surgical site mean that the procedure can be conducted safely. We hypothesize that this fixation technique reduces the frequency of micro-fractures of the wires in the electrode due to the micromovements, and that it minimizes iatrogenic intracochlear damage due to electrode manipulation after insertion, which could result in better hearing outcomes from robotic cochlear implantation.

## Background of Cochlear Implantation

### Surgical Techniques

During manual cochlear implantation surgery, the receiver-stimulator is usually fixated either with the standard bone recess and bony tie-down suture technique, or the tight subperiosteal pocket technique with or without a bone recess (5–9). In addition to that, there are fixation techniques using screws, meshes, bridges, and pins or pedestals (10, 11).

To prevent electrode migration, the electrode itself is often stabilized with various techniques. Electrode migration refers to any movement of the electrode array relative to its initial position within the cochlea at some point in time after surgery. Placing the electrode in channels within the mastoidectomy cavity in an S-form is recommended, completely below the bone surface, while bony overhangs should be kept to prevent extrusion. Furthermore, small hooks, open bony bridges, and bone paté or bone wax over a channel are used (7, 8, 12).

At the site of cochlear insertion the electrode is kept in place with a tight packing of tissues such as fascia, muscle, fat or with fibrin glue (13). Others create stabilizing grooves in a corner of the facial recess or split-bridges in the incus buttress (14–17), which has been shown to decrease electrode migration rates significantly (16). For this, Leinung and Loth et al. proposed an about 3 mm long groove with a diameter of 1.1 ± 0.05 mm with an opening of 0.9 ± 0.05 mm to secure a 1.3 mm electrode lead, creating holding forces equivalent to another fixation technique using a titanium clip on the posterior buttress (18, 19). Further techniques described suturing the electrode to the incus buttress or the posterior canal wall (20, 21).

### Cochlear Implantation Complications

Cochlear implantation has an average revision surgery rate of about 7.6% (22) and the device failure rate as recorded in clinics is about 5.1% (22). In the patient's timeframe, revision surgery rate has been measured as 1.0–1.9% per year (22–24). However, revision and device failure rates vary greatly between clinics with reported rates between 1.2 and 15.1%, and 0.5 and 14.7%, respectively (22), suggesting a great influence of other factors such as surgeon experience, the used surgical technique, or improved device reliability of new generations of implants (25).

Failed electrodes, most likely due to breakages of the thin wires, were found in 5.6–9.0% of devices (26–29). Furthermore, 54% of all devices had deliberately deactivated electrodes through reprogramming (27). As such, these failures did not appear to cause a decline in performance, however devices with more than three electrode failures were at high risk for future device performance deterioration leading to explantation (26, 30). The causes for these failures included loss of hermetic seal, fatigue fractures due to micro-movements in the electrode lead exit or the electrode lead itself (31–34), or external trauma, where the latter was reportedly more common in children (32, 35). In a recent revision case of robotic cochlear implantation, Morrel et al. observed wire fractures at the acute turn from the mastoid surface into the drilled tunnel (36), and have suggested smoothing the edges of the tunnel at the surface of the mastoid.

Electrode migrations can result in outcome decrement, due to insufficient stimulation of the low-frequency regions in the cochlear apex, and can also cause pain, vertigo, tinnitus or nonauditory stimulation (37, 38). A displacement of 3 mm results in a tonotopic change of about one octave (39), but the change in audiologic characteristics can usually be corrected through signal remapping (40). Studies have suggested a lower incidence of electrode migrations in perimodiolar electrode arrays than in straight lateral wall arrays (37, 41–43). However, perimodiolar have been found to have a higher incidence of primary scala vestibuli insertion or scala tympani to vestibuli translocations, and probably slightly higher incidence of tip fold-over (43). Both of these findings might be more related to surgical technique than the distinction between straight and perimodiolar array (43). Revision surgery caused by electrode migration is rare at around 0.2–2.5% (23, 42–48) per cochlear implant surgery in clinics, but electrode migration itself could have a much higher incidence. With various imaging modalities, electrode migration rates (at least one electrode out of the cochlea, or displacements >1 mm) have been reported anywhere from 0.4% in a direct postoperative scan (49), 7.4% 1 month after activation only in cases with an impedance increase (41), 13.4% at least 1 month after surgery (50), 29% after a mean follow-up time of 24 months (39), to 61% after a mean follow-up time of 34 months (51).

### Background of Robotic Bone Removal

Robotic surgical systems for bone removal in neurosurgery and otology have been studied in the past.

Federspil et al. created recesses for cochlear implants with a six degrees-of-freedom industrial robot arm in human *ex-vivo* specimens. Patient registration was performed by recording three points on the bone surface with a tracked tool, while the patient was fixated rigidly. Their optimal milling parameters were 30,000 revolutions per minute (RPM) spindle speed, a feed forward rate of 5 mm/s for calvarium, and 1 mm/s for mastoid bone. Furthermore, to maintain physiologically sparing temperatures of the bone during milling, the spiral path was preferable (52, 53). In a later work with the same robot and the same application, Stolka et al. presented an intraoperative method to generate bone surface meshes for planning through tracked ultrasound measurements with a reconstruction precision of about 0.7 mm, and a final implant bed precision of about 1 mm (54).

Korb et al. eliminated a lesion in the petrous bone in a clinical study on one patient with an image-guided serial robot arm. Patient-to-image registration was performed through four fiducial screws. Their cranial fixation system consisted of a vacuum mouthpiece-based fixation coupled with vacuum cushions, thus allowing for a non-invasive fixation. The measurement attempt of the end-to-end accuracy was 0.66 ± 0.2 mm, and a maximum deviation of 1.06 mm. The researchers noted that the use of an adapted industrial robot would hardly be possible in routine surgical interventions, mainly due to the necessary technical, logistic and regulatory constraints to be overcome. Furthermore, preoperative planning times of half an hour to an hour would be incompatible with clinical reality, so any new concepts must rely on fast and semi-automatic intraoperative planning (55).

Danilchenko et al. performed autonomous robotic mastoidectomies using an image-guided industrial six-axis robot arm in human *ex-vivo* temporal bones. Patient-to-image registration was performed through four fiducial screws. They used a feed rate of 1 mm/s and reported maximum errors of 0.6 mm. They also state that while the fundamental engineering concepts were well developed, the translation into clinics was less well studied, in particular issues around maintenance of sterility, transportation and setup of the system in the operating room and safety considerations (56).

Dillon et al. demonstrated robotic mastoidectomies and access cavities to the vestibular system with a four degrees-of-freedom compact skull-mounted robot in human *ex-vivo* specimens. Patient-to-image registration was performed through a positioning frame on fiducial screws. They reported an average surface border error of 0.38 mm, and standard deviations ranging from 0.13 to 0.39 mm for the mastoidectomy, and a root mean squared surface accuracy between 0.23 and 0.65 mm for the access to the vestibular system. While they could show that a compact bone-attached robot can efficiently perform bone-removal, they stated that translation of this approach to clinical use would face additional challenges (57, 58). Dillon et al. showed that to avoid relatively large transient forces, the burr should be kept as perpendicular to the bone surface as possible. Further, shallow cuts with larger velocities were better in terms of forces than deeper cuts with slower velocities (59).

## Materials and Methods

### Concept

**Figure 1:**
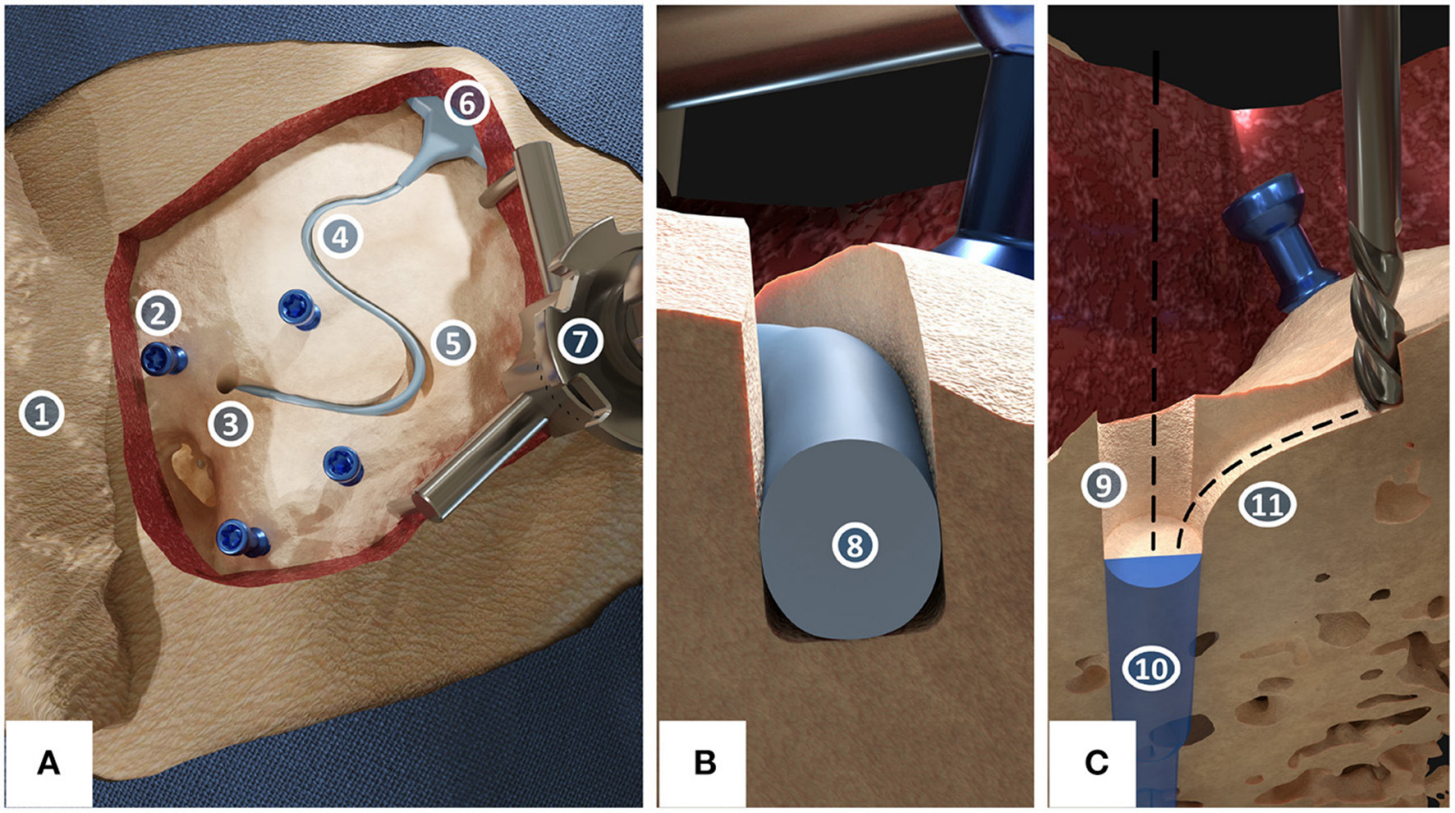
The proposed concept for robotic milling of electrode lead channels during robotic cochlear implantation. **(A)** Surgical site of a left ear with its pinna (1) after the incision and placement of four fiducial screws (2) in which center the middle ear access hole (3) has been drilled. The electrode lead lies within the milled channel (4) and channel widenings (5) while the receiver-stimulator (6) is placed in a subperiosteal pocket. The patient marker attachment tripod (7) is fixed to the skull using a fifth screw. **(B)** Cross-sectional view of the immobilization of the electrode (8) in the channel with a slight press fit. **(C)** Cross-sectional view of the access point preparation (9) for the planned middle ear access drill hole (10). The sharp edge between the drill hole and the channel has been rounded off to create a smooth tunnel-to-surface transition (11).

The proposed technique for the electrode lead management during robotic cochlear implantation foresees the creation of a channel without self-crossings on the surface of the temporal bone, starting in the middle ear access tunnel, and leading to a ramped bone recess for the electrode lead exit of the receiver-stimulator (Figure 1A). Insertion depth of the electrode array into the cochlea can deviate slightly from planning, changing the amount of surplus electrode lead to be stowed below the temporal bone surface. Thus, one or more widenings of the channel are introduced (Figure 1A). Widenings provide space to accommodate a range of different paths within, resulting in different lengths of the electrode lead stowed.

#### Standardization and Reproducibility

The image-guided planning and robotic execution coupled with a software-guided clinical workflow creates a standardized and reproducible method for electrode management during robotic cochlear implantation.

#### Prevention of Iatrogenic Intracochlear Damage

Using the image-guided robotic approach, the electrode lead channel is milled before insertion. Thus, after insertion the electrode lead is first fixated in the press fit channel at the tunnel-to-surface transition. From then on, further movements of the electrode lead on the surface will not transfer to the electrode array in the cochlea, thus prohibiting further iatrogenic intracochlear damage.

#### Consistent Protection of the Electrode Lead From Mechanical Fatigue Due to Micro-Movements

To prevent fatigue fractures due to micro-movements, the channel is milled with a cylindrical milling cutter that is slightly smaller than the electrode diameter, creating a press fit that keeps the electrode lead immobilized in a stable fixation (Figure 1B). To further avoid the sharp angle between the middle ear access tunnel and the mastoid surface, a rounded path is milled to create a smooth tunnel-to-surface transition (Figure 1C), as suggested by Morrel et al. (36). The rest of the channel is also curvature-optimized to achieve a fixation for the electrode lead requiring minimal bending. Milling the electrode lead channel before the middle ear access tunnel presents the opportunity to shape the potentially inclined cortical bone surface at the access point to the tunnel into a flat surface to provide for optimal conditions for the high-accuracy requirements of drilling through the facial recess (Figure 1C).

#### Consistent Protection of the Electrode Lead From External Trauma

Using an image-guided approach, protection of the electrode lead from trauma can be ensured through the creation of a channel at a uniform and sufficient depth below the bone surface. The press fit design of the channel will hold the electrode lead below the bone surface.

#### Consistent Protection of the Electrode Lead From Electrode Migrations

Similar to other fixation techniques, the electrode lead will be fixated on the temporal bone surface with a press fit channel and constrained in the drill tunnel toward the entrance to the cochlea, thus prohibiting electrode migration.

### Workflow in the Clinic

**Figure 2:**
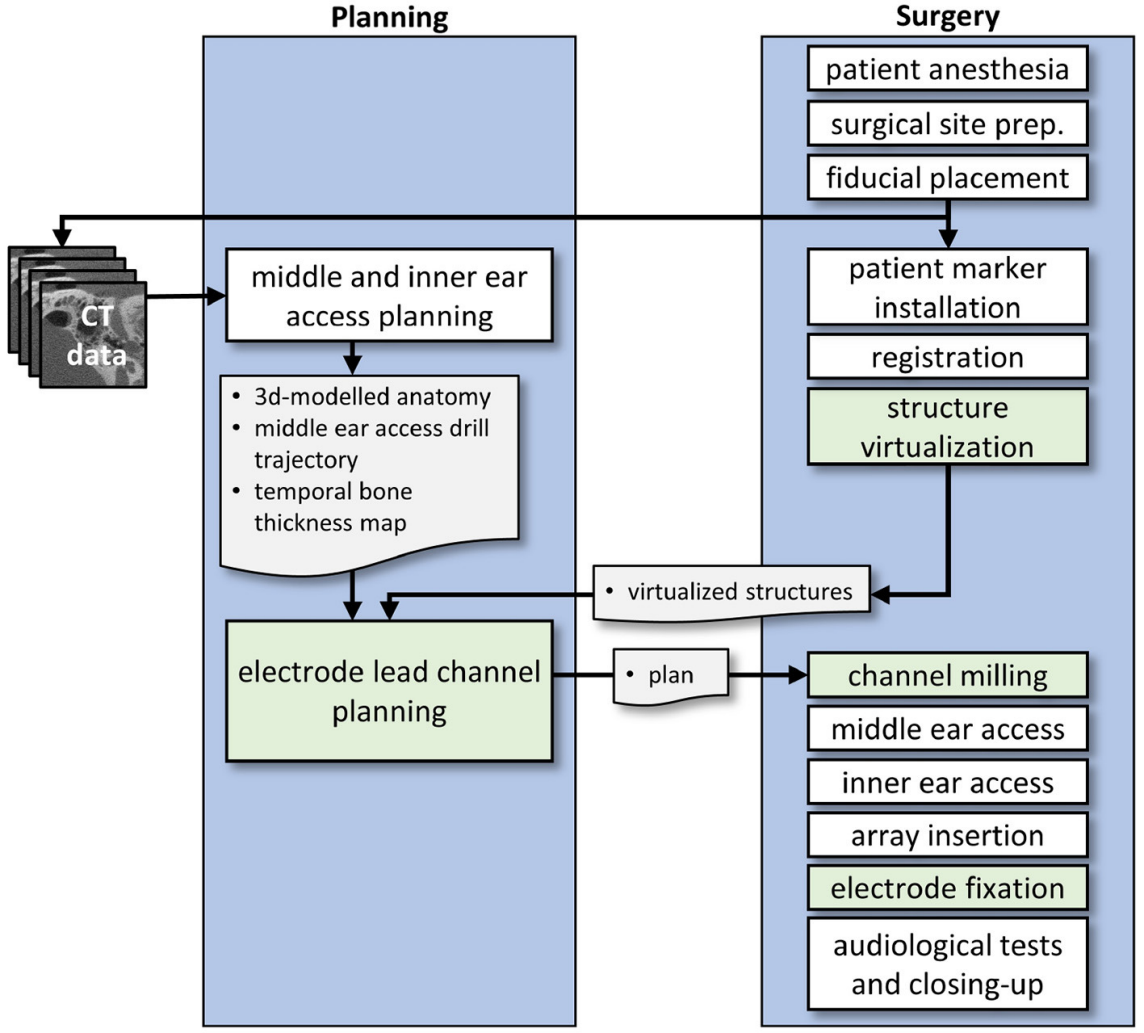
Conceptual workflow of the proposed electrode lead management technique. During middle ear and inner ear access planning, there is a possible concurrency when the structure virtualization can be completed. The results of both processes are required for electrode lead channel planning. Workflow elements that are developed and investigated in this work are marked in green color.

A clinical workflow for robotic lead channel milling during robotic cochlear implantation was developed (Figure 2).

First, patient anesthesia is performed and the surgical site is prepared. A retroauricular incision is created, in our experiments with a lazy-S incision. A tight subperiosteal pocket and flat surface, or a ramped bone recess for the receiver-stimulator is created, and a receiver-stimulator mock-up is inserted and tested for good fixation. Same as during the manual surgery, the surgeon chooses the location based on the local anatomy (e.g., flat and smooth bone surface), and considers the necessary distance from the pinna, such that the external audio processor will not interfere with the internal receiver-stimulator. Surgeons consider glasses, aesthetics like the visible bump from the internal device, and the hairline. Another possibility is the virtual mirroring of the contralateral implant in bilateral cases.

After the preparation of the receiver-stimulator fixation, five fiducial screws for robotic cochlear implantation are placed. High-resolution medical images (e.g., CBCT scan) are acquired and used for the planning of the robotic procedure. Once the relevant anatomy has been segmented (i.e., temporal bone, the incus and malleus, the stapes, the facial nerve, the chorda tympani, the cochlea and its bony overhang, and the sigmoid sinus), and the cochlear parameters as well as the cochlear duct length measured, the middle and inner ear access can be planned.

In the meantime, the patient marker is attached to the skull using one of the screws. Patient-to-image registration is performed by digitizing the four other fiducial screws using a screw-specific tracked registration tool. For the planning of where to create the electrode lead channel, the accessible temporal bone surface must be known. Thus, the user is guided through the steps of virtualizing structures of the surgical site. The skin incision borders are mapped with a tracked tool, as well as the attachment of the patient marker. The position of the receiver-stimulator is recorded with the tracked tool at this point, or, if a contrast-enhanced version of the receiver-stimulator had been used, it is automatically detected by the navigation software.

The electrode lead management is planned. Now that all relevant structures have been mapped and visualized in the virtual patient anatomy, the channel path can be defined on the surface of the temporal bone model. The length of the channel is calculated from the position of the receiver-stimulator relative to the entrance of the middle ear access drill hole, the length of the drill hole until the round window, and the length of the electrode lead of the chosen cochlear implant. For the insertion depth, full insertion is assumed when no reasons for a partial insertion are detected (e.g., cochlear ossification). Curvatures are required to be below a threshold where the electrode might be damaged. The robotic execution shall not collide with other structures present in the surgical site. With the planned middle ear access trajectory, the insertion depth, and the position of the receiver-stimulator determined, the excessive lead length on the surface can be calculated up to intraoperative uncertainties. A safety margin of 1.0 mm is respected from the planned channel to surrounding structures such as dura mater, sigmoid sinus, external auditory canal, the skin incision, as well as to the structures necessary for the image-guided surgery, namely the fiducial screws and the patient marker attachment.

The lead channel milling is then executed under constant irrigation. The image-guided robot mills the channel along the planned path while controlling the feed-forward speed based on force measurements and navigation errors.

In the next steps, the rest of the robotic cochlear implantation workflow is executed, as described by Weber et al. (1, 60–62). This incorporates the middle ear access, the inner ear access, and the insertion of the cochlear implant electrode array through guiding metal half-tubes, which are removed as soon as the insertion is complete.

Lastly, the electrode is embedded in the milled channel beginning in the tunnel-to-surface transition, then from the receiver-stimulator toward the widening where the rest of the excess lead is stored. The surgeon closes the wound as soon as the audiologist has successfully tested the functionality of the implant.

### Software Pipeline for Planning of Electrode Lead Channels

**Figure 3:**
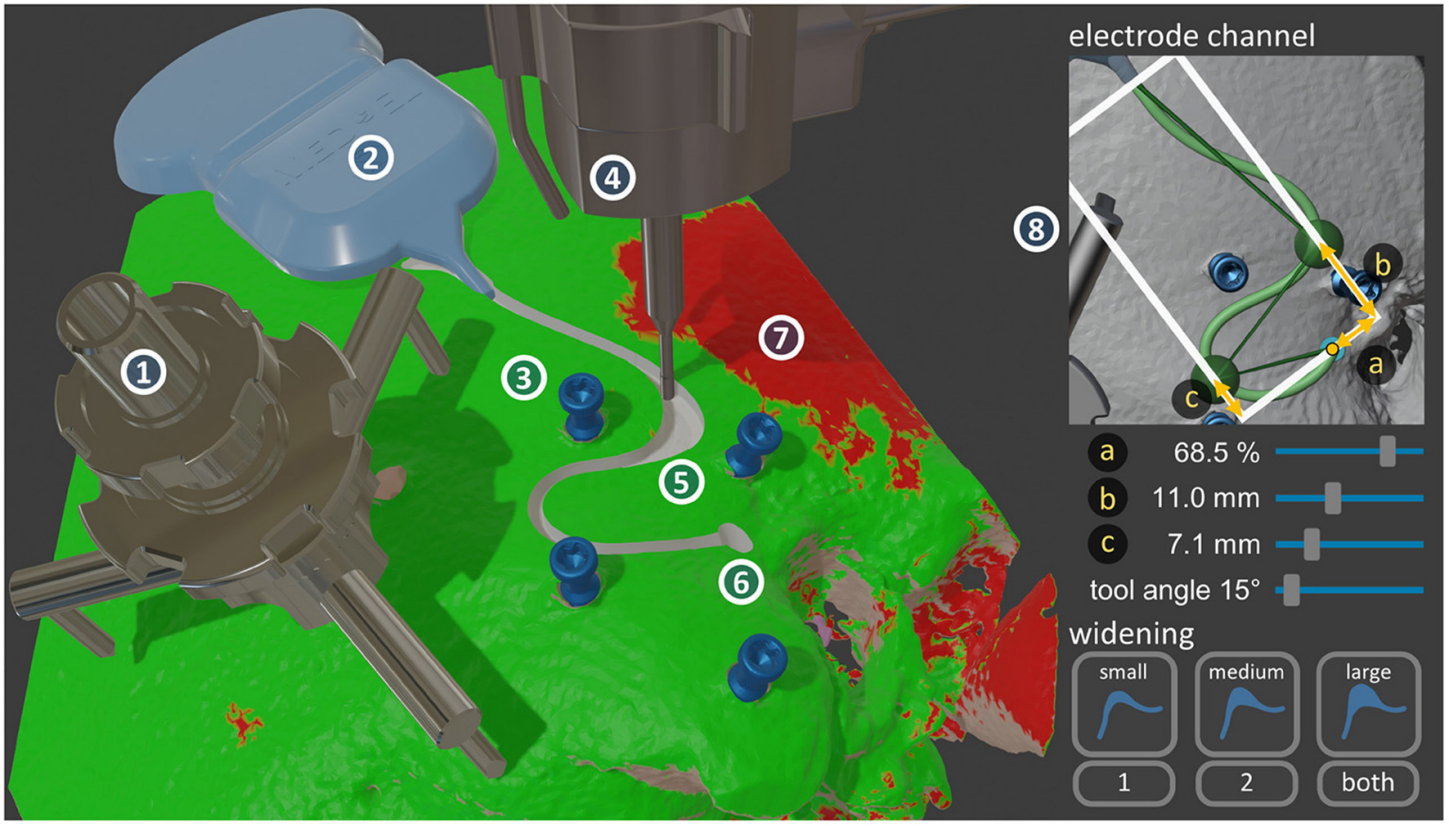
Left: The custom-developed surgery planning software, implemented in Blender. The visualized objects are the HEARO Patient Marker Attachment tripod (1), the silicone template of the receiver-stimulator (2), the four HEARO Fiducial Screws (3), the end-effector with the milling cutter (4) in the planned electrode lead channel widening (5), and the planned drill access hole to the middle ear (6). All these structures reside on the patient's reconstructed temporal bone, onto which a red-green map is overlayed showing where the bone thickness is sufficient to place a channel plus safety margin (7). Right: A possible user interface implementation for the planning workflow (8).

A prototype of a surgery planning software was developed as an add-on to the computer graphics software Blender (Blender Foundation, Amsterdam, Netherlands). This planning add-on (Figure 3) allows the loading of the patient case from the otologic planning software OTOPLAN (CASCINATION AG, Bern, Switzerland), containing the information about the middle and inner ear accesses, and all the reconstructed anatomical structures (i.e., temporal bone, the incus and malleus, the stapes, the facial nerve, the chorda tympani, the cochlea and its bony overhang, and the sigmoid sinus). It displays the previously virtualized structures, that is, the receiver-stimulator, tripod attachment of the patient marker, the patient marker itself, and the skin incision borders.

#### Virtualization of the Surgical Site

The position and orientation of the receiver-stimulator was obtained by recording first the end of the electrode lead exit, the fantail, then the two other points on the triangle shape of the fantail in a counter-clockwise fashion. On the tripod, equidistant recording of the three legs was sufficient to define its pose. The skin incision was virtualized by recording individual points along its border. With this plus the overlayed thickness map onto the temporal bone, the available areas for safe electrode channel milling were known.

#### Access Point Preparation

This part of the milling path was calculated automatically with the information about the middle ear access trajectory. It was milled in levels of 1.0 mm to a depth of 5.0 mm by first plunging down in the middle in the orientation of the trajectory, then creating the cylindrical shape by following a circle around the trajectory axis.

#### Electrode Channel

The channel path was expressed as three connected Bézier splines, creating an overall path with two curves. The ends were fixed at the position of the receiver-stimulator electrode lead exit, and the middle ear access trajectory entrance. First, the shape of the two curves was chosen, where the choice is between an S-shape that first starts in the posterior direction, or the mirrored S-shape that starts in the anterior direction. The position of the two curves was determined with two control points. Three input sliders determine the channel shape, two controlling the distance of the two control points from the entrance in the direction of the receiver-stimulator, and one the offset of the two control points in the perpendicular direction (Figure 3). The remaining degrees-of-freedom were used to optimize the channel shape for low curvatures in all turns, and to achieve the required length. The navigation software warned the user if the calculated channel path had curvatures exceeding a critical threshold (i.e., a curvature radius of 5.0 mm). Another slider controlled the angle of the milling cutter relative to the middle ear access trajectory orientation around the axis in-between the receiver-stimulator and the drill hole entrance. This enabled the tilting of the milling cutter away from structures such as the patient marker attachment. The display of the channel's safety margin allowed the visual confirmation that the channel will not collide with any structures in the surgical site. Additionally, a map showing areas of sufficient thickness for the placement of a channel could be overlaid onto the temporal bone (Figure 3). The channel depth was automatically calculated to be at a uniform distance below the bone surface along the whole length, to protect the electrode lead from external impacts.

#### Tunnel-to-Surface Transition

This transition from drill tunnel to electrode channel was calculated automatically with a curvature-optimized Bézier spline in-between the already defined access point preparation path and the electrode channel path.

#### Widening

The location of the widening could be chosen in either the first curve, the second curve, or in both (Figure 3). The other choice was the size of the widening (e.g., small size was 1.0 mm shorter, 2.0 mm longer than the planned length). The boundaries of the widening were achieved by finding two tangent Bézier splines to the curve that satisfy the length requirements. Then, the milling path for the widening cavity was calculated through iterative contour-offsetting of the widening shape with a path overlap of 70%, then connecting the resulting paths in an outwards-spiral fashion. This spiral pattern had been previously suggested by Federspil et al. (52). Additionally, climb milling (also called down-cutting) was planned where the cut of the bone chips is started at the maximal width, as different from conventional milling (i.e., up-cutting) since this generates less heat (63).

#### Implant Bed

With this implementation of a planning software, only a ramped bone recess for the fantail was milled. Same as with the widening, the milling path was calculated by taking the outer contour of the fantail, offsetting this contour and then connecting the resulting paths in a spiral fashion. There was a possibility to add pin holes for receiver-stimulator versions with pins.

### Adaptation of a Surgical Robotic System

**Figure 4:**
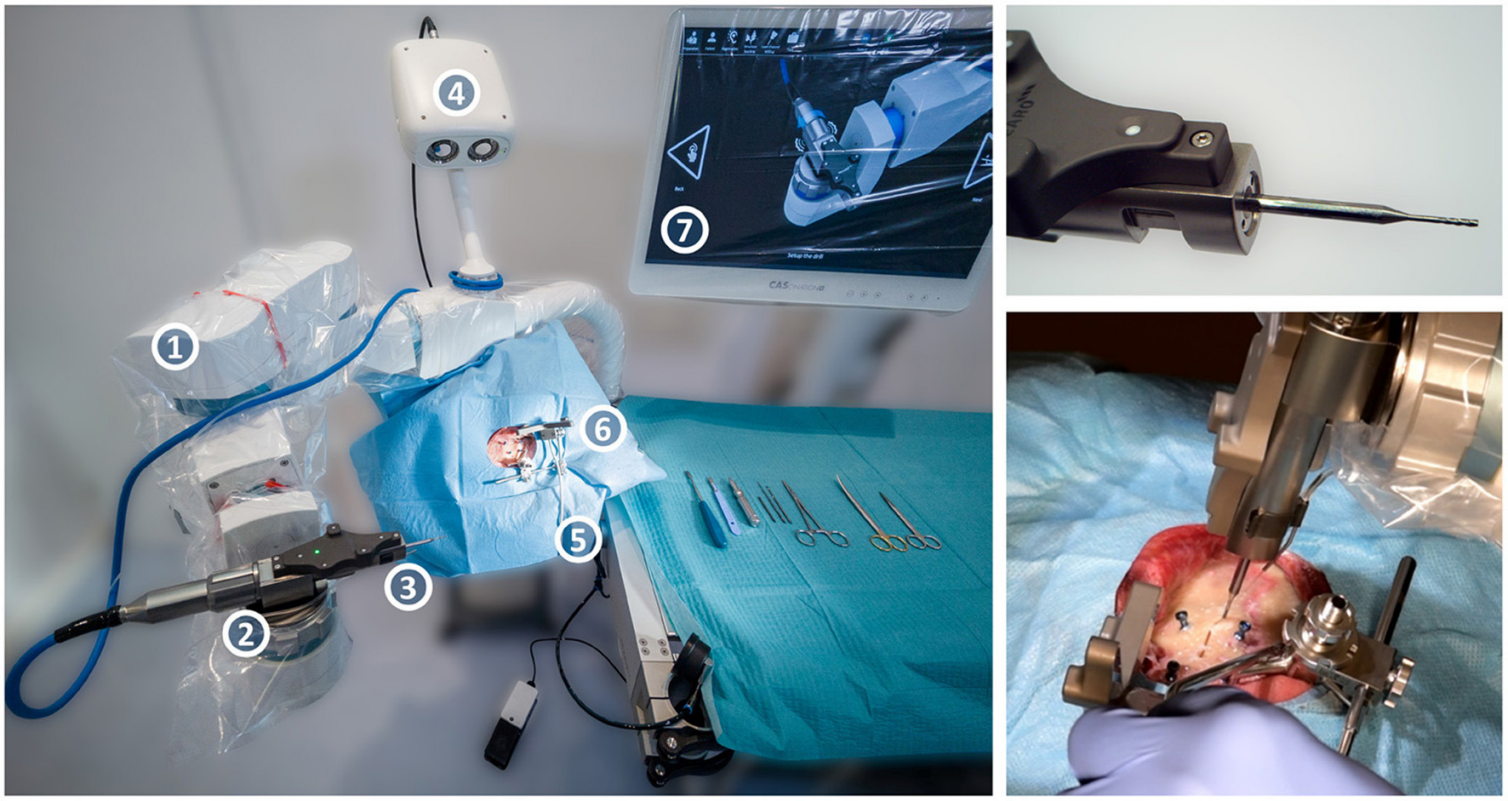
The HEARO robotic surgical system, consisting of the five-axis HEARO Robot (1) with a tracked HEARO Drill end-effector on the quick-release wrist mount (2), the cylindrical milling cutter (3), a high-precision tracking camera (4), and a carbon-fiber headrest with air-pressure cushions (5) under a draped specimen with a patient marker attached (6). The navigation software displayed on the draped screen guides the surgeon through the procedure (7). On the top right, a close-up of the end-effector with the milling cutter, and on the bottom right the robotic system in the process of milling a channel during the *ex-vivo* study.

For the execution of the experiments, the commercially available robotic surgical system HEARO (CASCINATION AG, Bern, Switzerland) for minimally invasive cochlear implantation was modified to enable electrode lead channel milling, instead of the middle and inner ear access drilling purpose for which it is intended (Figure 4). The HEARO Step Drill 1.8 mm was used to create the middle ear access drill hole. The technology and function of the system has been described by Weber et al. (1).

The modifications to the system include a HEARO Drill end-effector capable of reaching high spindle-speed of up to 80,000 RPM with an external motor controller and power supply, a custom-developed H10F tungsten carbide 1.2 mm diameter cylindrical three-fluted milling cutter with a center tap, and the necessary software modifications to adapt the system to milling purposes.

### Workflow of the Experiments

An experimental study on formalin-flushed full-head human *ex-vivo* specimens with the approval from the local ethics committee (KEK Bern, Switzerland, Project-ID 2018-00770) was conducted to determine the safety and efficacy of the proposed approach.

The experimental study was conducted as follows: first, the robotic system was set up, and the end-effector calibrated. Then, the robotic channel milling, middle ear access and inner ear access were executed as described above.

The milling parameters were chosen as follows: an approximately perpendicular inclination to the surface normal, with spindle speeds of 30,000 RPM at a depth of 2.3 mm with a forward velocity setpoint of 2.0 mm/s, and force-based linear feed-forward velocity control above 4 N up to a threshold of 10 N, where the system interrupts the procedure and asks for user interaction. These milling parameters were previously determined in a pilot study based on milling forces, channel accuracy, navigational errors, and the drawn current by the end-effector (64). Each specimen was milled with a separate milling cutter. The introduced channel widening was designed to allow deeper insertions into the cochlea than planned by 1 mm, and shallower insertions by 2 mm. The location of the widening was chosen either in the first curve or in the second curve, based on the anatomy.

The cone-beam computed-tomography (CBCT) images for surgery planning were taken with an xCAT XL mobile head scanner (Xoran Technologies LLC, Ann Arbor, MI, USA) with an X-ray tube voltage of 120 kV, a tube current of 7 mA, and an isotropic reconstruction resolution of 0.1 mm.

For the planning of the robotic surgery on these images the otologic planning software OTOPLAN Version 1.5 (CASCINATION AG, Bern, Switzerland) was used. This software supports the generation of patient-specific 3D reconstructions of the anatomy, and then allows planning of the middle and inner ear access for robotic cochlear implantation.

The robotic execution was followed by the excision of the temporal bone from the whole-head specimen, and the acquisition of a micro-CT scan at an external certified testing laboratory (units MITTELLAND AG, Zuchwil, Switzerland), using a calibrated (sphere distance difference smaller than 4 μm) industrial computer tomograph (Metrotom 800, Carl Zeiss IMT GmbH) with an isotropic reconstruction voxel size between 40 and 50 μm. Once returned, the insertion of the electrode array was performed on the temporal bone by G.B., an otorhinolaryngology surgeon with cochlear implantation experience. For this, a silicone mock-up of the MED-EL Synchrony 2 Mi1250 FLEX28 cochlear implant was used. Subsequently, photos of the embedded electrode were taken, and the following endpoints measured in the gathered data.

### Evaluation of Safety and Efficacy

We hypothesize that robotic electrode lead channel milling (planning and execution) can provide for an electrode lead channel that immobilizes the electrode lead and protects it from trauma. This can be further split into the following hypotheses: a channel for a cochlear implant electrode can (a) be robotically milled such that it remains within a planned safety margin, (b) be planned and executed such that postoperatively, the whole length of the electrode lead can be embedded within the channel, (c) be robotically milled such that it will create a slight press fit, designed to prohibit micro-movements, and (d) be planned and executed such that the depth is always greater than the electrode diameter.

The primary endpoint was the rate of completely milled lead channels, where the surgeon was able to immobilize the electrode lead without further manual milling, excluding the potential milling of an implant bed for the receiver before robotic execution. The secondary endpoints were split into subcategories safety and efficacy.

In terms of safety, the following measurements were carried out.

#### The Lateral Displacement and the Depth Displacement

**Figure 5:**
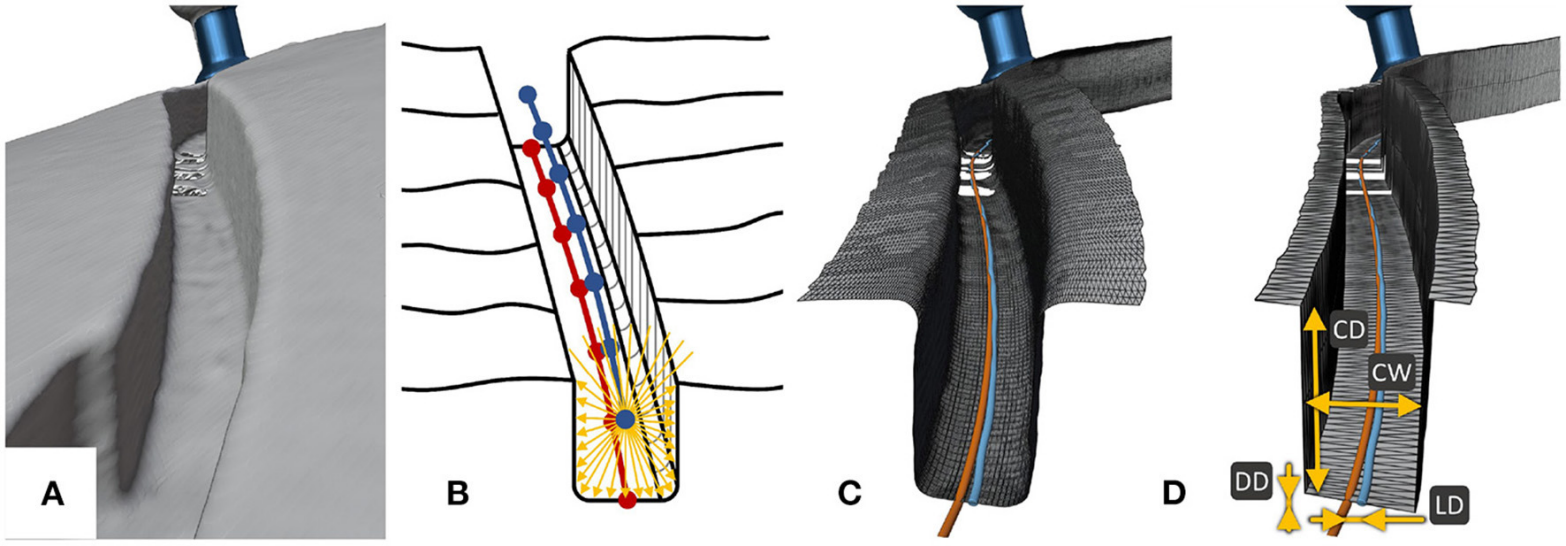
The process for the analysis of the milled channels. **(A)** The reconstructed surface of the resulting electrode channel from the micro-CT. **(B)** This reconstructed surface was sampled radially to the approximate channel center line, resulting in **(C)** a resampled surface containing only the channel, which was then classified into top left, top right, left wall, right wall and bottom. **(D)** The measurements through these classifications were displayed as a simplified version of the channel, where CD stands for channel depth, CW for channel width, DD for depth displacement, and LD for lateral displacement.

The measurements were taken in the micro-CT scan on the reconstructed temporal bone surface (Figure 5A). For this, the temporal bone was segmented using a medical image analysis software (Amira, Thermo Fisher Scientific, Waltham, Massachusetts, USA), then postprocessed manually using the computer graphics software Blender. First, the co-registration of the postoperative micro-CT with the preoperative CBCT was carried out through paired-point matching of the positions of the four fiducial screws. Secondly, the approximate resulting center line of the channel was estimated, which ideally should correspond to the planned center line, that is, the milling path (Figure 5B, red line). Then, from each point along this approximate resulting center line (Figure 5B, blue line), the surrounding channel walls were mapped perpendicular to the path using ray intersections with the 3D reconstruction models. This resulted in a mapped version of the bone surface of connected cross sections (Figure 5C). From the acquired points, the corresponding normal vectors, and their order, the channel walls can be classified into top left, left, bottom, right, and right top. Finally, the measured dimensions of the resulting channel were calculated by averaging the positions of the classified groups. The resulting center line is the line in the middle between left and right wall on the bottom (Figure 5D, blue line). The measurement results of the channel walls could then be displayed as a simplified channel model (Figure 5D). Measurements were split into lateral and depth directions based on the planned milling cutter orientation. In the widening, the right and left walls were treated separately. Air cells were removed from the reconstructed bone surface manually from the three-dimensional mesh, using the information of both the CBCT from planning and the micro-CT images. The resulting gaps in the channel were treated as missing data, and hence did not influence the measurements.

#### The Length of the Resulting Channel Outside the Safety Margin

This was measured manually by visual inspection in the postoperative micro-CT with the co-registered plan. The safety margin around the channel was overlayed transparently, and visually checked for an intersection with the resulting channel.

In terms of efficacy, the following measurements were carried out.

#### The Channel Width and the Channel Depth

The analysis from lateral and depth displacement was used. The channel width was measured as the distance between the left and right wall of the resulting lead channel, whereas the channel depth was measured as the height difference from the mean of the surface on the top left of the channel and the top right of the channel, to the bottom surface of the resulting electrode lead channel.

#### The Length of the Electrode Lead Outside the Resulting Channel

The insertion depth was checked visually by the surgeon (full insertion, partial insertion), and the length outside the channel was measured manually with a ruler during the experiments and was visualized in the photos taken.

### Sample Size

The sample size was calculated based on a power analysis on a one sample variance and based on two experiments of a pilot study (64). One experiment had been conducted in bovine cortical bone, where 30 straight channels of various depths had been milled with three separate registrations, for a total of 717 mm milled channel length. The second experiment had been conducted in two preliminary cases with human *ex-vivo* temporal bones, resulting in a milled channel length of 44 mm. The root mean square errors for lateral displacement, depth displacement, channel width, and channel depth were 0.13, 0.17, 0.05, and 0.21 mm, respectively. Electrode lead channels for the implant used in the experiments (MED-EL Synchrony 2 Mi1250 FLEX28 Electrode Array) are about 60 mm long. The measurements were assumed to be independent from each other after the length of the tool diameter, so every 1.2 mm. Experience with drilling shows no systematic errors, but the patient-to-image registration does theoretically add a bias per case. However, over all cases the average bias was centered around zero.

For the endpoints of efficacy (i.e., channel width and channel depth), the risk threshold, that is the probability of violating the safety margin, was set to 1% per patient. For the endpoints of safety (i.e., lateral and depth displacement), the probability was set to 0.01% per patient, meaning that only in about one in 10,000 patients a safety violation would occur. Since the safety margin for the endpoints of safety are 3D reconstructions from a CBCT scan, their reconstruction error also had to be considered. According to Rathgeb et al. that segmentation error was 50 ± 50 μm in mean and standard deviation using the same technology (65). Using the binomial distribution, the probability for the hypothesis test can be calculated for one measurement point. Thus, to assume that the system can perform the tasks safely, the probability of going beyond the safety margins in one measurement point would need to be smaller than 0.0002% for safety endpoints, and 0.0201% for efficacy endpoints. For a safety margin of 1.0 mm, the corresponding root mean square error is 0.22 mm for the safety endpoints, and 0.28 mm for efficacy endpoints. For the power analysis, we use an alpha (Type I error) of 0.1%, and a power (Type II error) of 95%. Using this information, the required sample size returned six specimens. For redundancy, to account for unavailable measurements when mastoid air cells are present, and to account for anatomical variety, we conducted this study on seven anatomically distinct calvaria, resulting in a total of 12 temporal bones. Two sides were excluded, one had already been used for other experiments, the other presented a fractured temporal bone at arrival in the laboratory.

## Results

**Figure 6:**
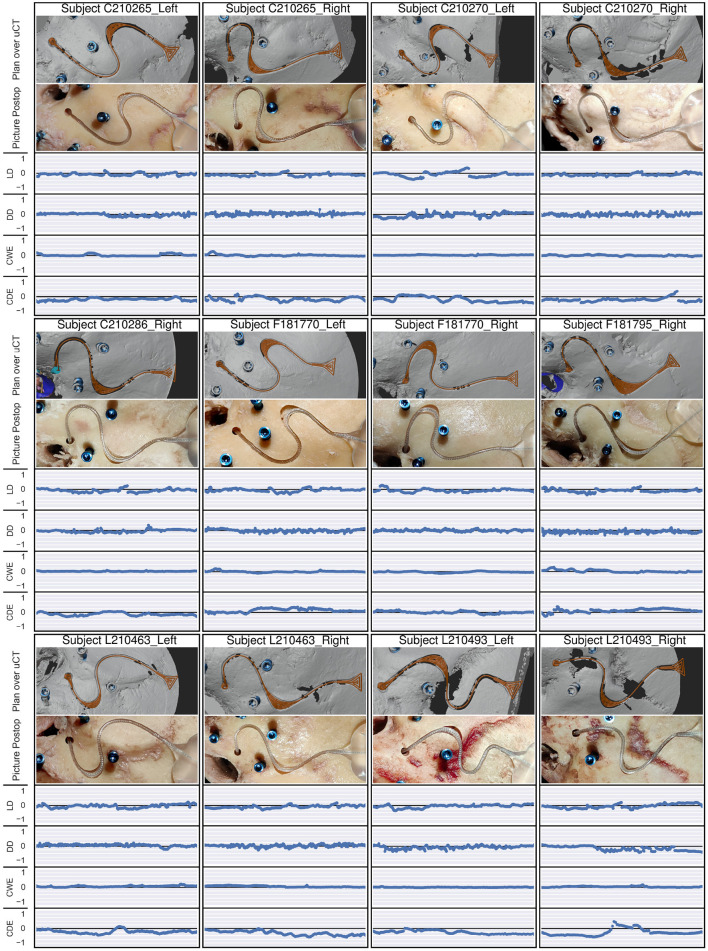
For each subject, the top image depicts the planned path (orange) over the reconstructed temporal bone surface from the micro-CT (uCT) scan, containing the resulting electrode lead channel. The second image is a photo of the postoperative result after electrode insertion. The four following graphs show the lateral displacement (LD), the depth displacement (DD), the channel width error (CWE), and the channel depth error (CDE), all on a scale from −1 to 1 mm, which corresponds to the chosen safety margin. The safety margins were respected in all endpoints and cases.

**Figure 7:**
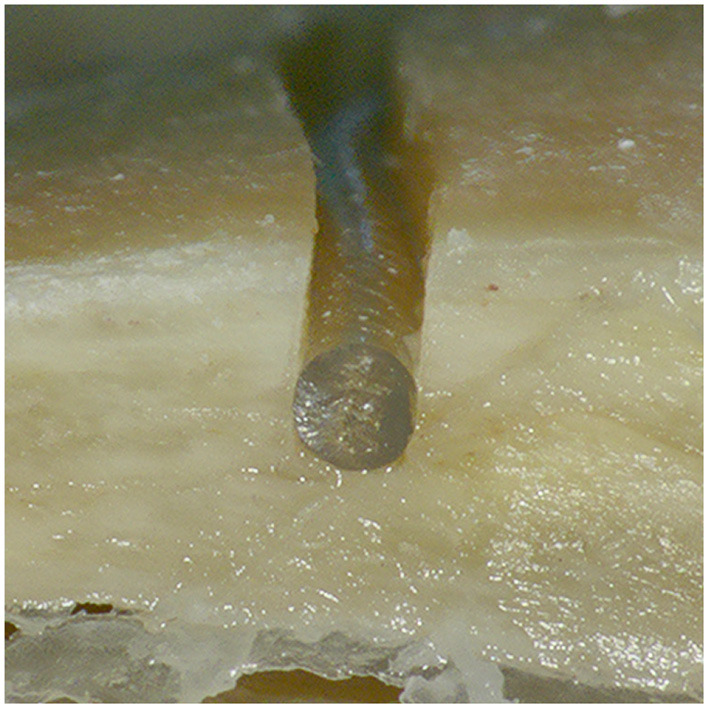
Cross section of the resulting channel with an embedded electrode lead cut for visualization. The electrode lead lies completely below the surface of the temporal bone and is held in place through the slight press fit between the channel walls.

**Figure 8:**
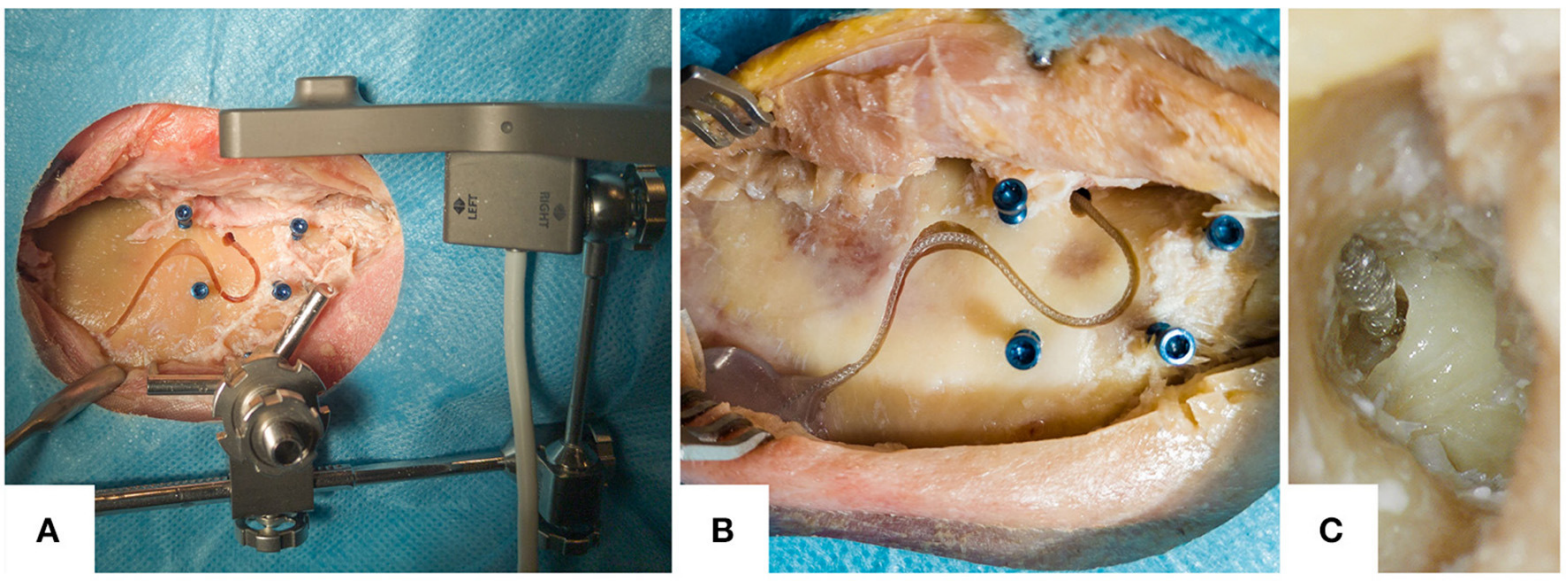
Example photos of the resulting electrode lead management. **(A)** The resulting electrode channel with the attached patient marker on the tripod attachment. **(B)** Close-up of the electrode lead embedded within the resulting channel, in another specimen. **(C)** A microscope image through the external auditory canal after the elevation of the tympanomeatal flap. It shows the tapered neck of the electrode array, which is being inserted into the cochlea at the round window.

All 12 out of 12 cases were successfully and completely planned and milled (Figure 6). All cases were completed with successful cochlear insertions. The electrode could be placed into the milled channel without having to force it, and when embedded was held through the slight press fit of the channel walls (Figure 7). In three out of the 12 cases, full insertion (i.e., the tapering stopper of the electrode array was close to the round window, as planned, seen in Figure 8) could not be achieved by 2–3 mm. However, in all cases all electrodes were within the cochlea since there is a distance between the tapering stopper and the first electrode. Nevertheless, the measured lead length outside the resulting channel was zero in all cases, so the full length of the electrodes could be embedded within the milled channel and the widening. After electrode array insertion, the surgeon could clip the electrode into the channel at the middle ear access drill hole entrance, thus allowing manipulation of the electrode lead without the electrode array being pulled out of the cochlea.

**Figure 9:**
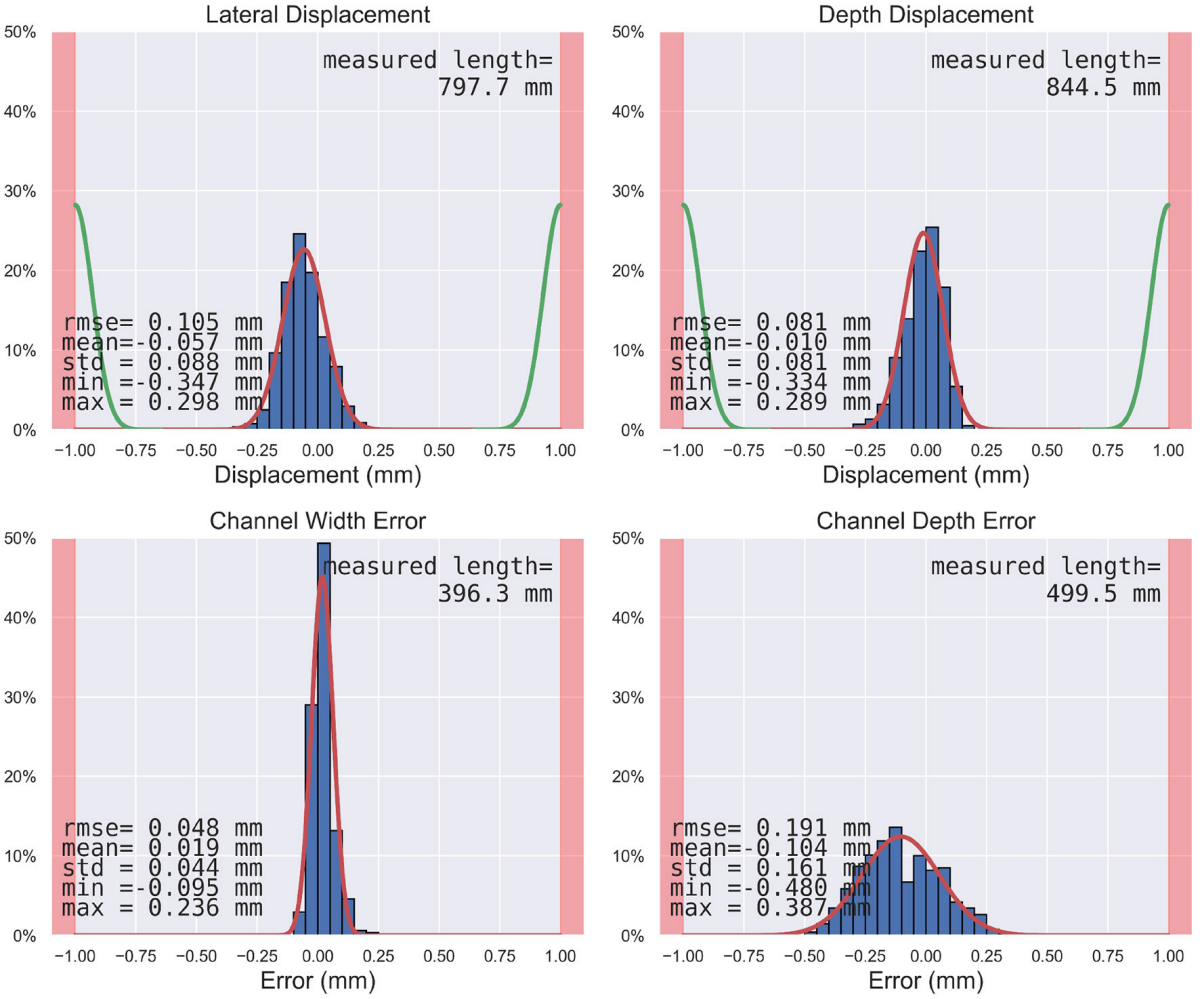
Histograms of the measured endpoints (i.e., lateral displacement, depth displacement, channel width error, channel depth error) in blue, the estimated distribution in red, the segmentation error distribution in green (65) where applicable, and the safety margins as red zones at the sides. The measured length is the length along which the individual measurements were carried out in the resulting channel and widening.

The resulting channel never exceeded the planned safety margin in any case. The mean lateral displacement was −0.06 mm [standard deviation (SD) = 0.09] with a root mean square error (RMSE) of 0.11 mm, where the negative mean value indicates that the displacement tended toward the left in the milling direction. The maximal absolute lateral displacement was 0.35 mm. The mean depth displacement was −0.01 mm (SD = 0.08), with maximal depth displacements deeper than planned of 0.33 mm, shallower than planned of 0.29 mm, and an RMSE of 0.08 mm. The maximal combined lateral and depth error was 0.42 mm. The limit RMSE for the admissible risk (i.e., leaving the safety margin of 1.0 mm with a probability of 0.01% in a patient) would be 0.22 mm. Since both lateral and depth RMSE are below this limit, including the 99.9% confidence interval (see Table 1), it can be stated that the channel for a cochlear implant electrode lead could be robotically milled such that it remained within a planned safety margin of 1.0 mm, while the risk of leaving that safety margin is smaller than one case in 10,000 (Figure 9).

**Table 1:** Summary of the measured endpoints.

	**Mean (mm)**	**Standard deviation (mm)**	**Root mean square error (mm) [99.9% CI]**	**Minimum (mm)**	**Maximum (mm)**
Lateral displacement	−0.057	0.088	0.105 [0.096, 0.115]		0.347
Depth displacement	−0.010	0.081	0.081 [0.075, 0.089]	−0.334	0.289
Channel width error	0.019	0.044	0.048 [0.042, 0.055]	−0.095	0.236
Channel depth error	−0.104	0.161	0.191 [0.171, 0.216]	−0.480	0.387
Electrode length outside the channel	0	0	0	0	0
Resulting channel length outside the safety margin	0	0	0	0	0

*For lateral displacement, the maximum is the maximal absolute deviation both to the left and right in the milling path direction*.

The electrode leads could be embedded within the channels and were immobilized by the slightly narrower channel as compared to the electrode diameter. The mean channel width was 1.22 mm (SD = 0.04), where the planned channel width and tool diameter was 1.20 mm and the electrode diameter was 1.30 mm. The mean channel depth was 2.20 mm (SD = 0.16) with a RMSE of 0.19 mm, and the planned channel depth was 2.30 mm. The minimal depth was 1.82 mm, still great enough to contain the full 1.30 mm diameter of the electrode used for the experiments. The limit RMSE for the admissible risk (i.e., leaving the safety margin of 1.0 mm with a probability of 1% in a patient) for these two efficacy endpoints is 0.28 mm, and thus higher than the measured error including the confidence interval.

**Figure 10:**
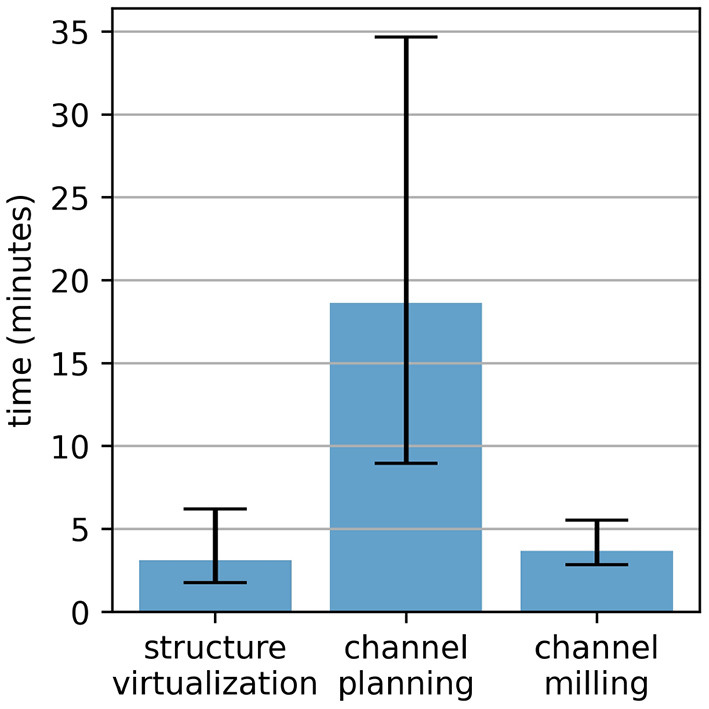
Time spent for the electrode lead management during robotic cochlear implantation in this *ex-vivo* study.

The individual steps of the proposed electrode lead channel management were timed (Figure 10). The additional step of virtualizing the structures of the surgical site (i.e., the receiver-stimulator mockup, the tripod attachment for the patient marker, the skin border) took about 3 min on average. Using the custom-developed planning software for robotic electrode channel milling, the planning time from case loading to the exported milling path ranged from 9 to 35 min with an average of 19. The time intervals from start to stop of the milling motor were between 3 and 6 min. During the experiments, milling forces usually ranged between 2 and 8 N with a maximal force of 10 N. Spindle torques were recorded between 1 and 7 mNm with a maximal torque of 8 mNm. An explanatory video showing the process from planning to robotic execution is provided as Supplementary Material.

## Discussion

In this paper, we have proposed a method for electrode fixation for robotic cochlear implantation. A possible clinical workflow was described, and an experimental study investigating safety and efficacy on *ex-vivo* human specimens was conducted using a custom-developed planning software and a modified commercial robotic surgery system. It could be shown that channels for cochlear implant electrode leads can be robotically milled such that they remain within safety margins of 1.0 mm. Also, the full length of the electrode leads could be embedded within the channels in all cases. The measured resulting channel width of 1.22 mm (SD = 0.04) with a tool of diameter 1.20 mm showed that this channel will create a slight press fit along most of the channel length, prohibiting micro-movements. Furthermore, the milled channel was never shallower than the full electrode diameter of 1.30 mm, with a minimal depth of 1.82 mm. With a channel depth of 2.30 mm and a safety margin of 1.0 mm, it was possible to plan a channel in all 12 cases by choosing a path in areas with sufficiently thick bone. Therefore, this approach could provide for a channel that could be planned and executed such that the depth was always greater than the electrode diameter, and that immobilizes the electrode lead and protects it from trauma.

The probability that the safety margins would be violated in safety endpoints was smaller than 0.01% or one in 10,000 (*p* < 0.001), and for efficacy endpoints smaller than 1% or one in 100 (*p* < 0.001). Thus, the procedure was shown to be safe in this *ex-vivo* model. Based on the new data, the safety margins could even be decreased to keep the same probabilities of going beyond the safety margins of 1% for the efficacy endpoints, and 0.01% for the safety endpoints. In that case, the margin for the safety endpoints could theoretically be as low as 0.7 mm, and 0.8 mm for the efficacy endpoints, thus enabling an increased possible patient population especially with thin skull thicknesses, or small anatomies.

Past research often stated that the presented surgical systems work well, but further questions would need to be answered to introduce them into routine care, such as sterilization, transportation from storage to operating room or space requirements (55, 56, 58). The experimental work in this study was conducted using a commercially available task-specific robotic device (HEARO, CASCINATION AG, Bern, Switzerland) that has fulfilled all necessary regulatory requirements.

In agreement with previous work, we have confirmed spindle speeds of 30,000 RPM to be sufficient for robotic milling (53, 56). However, in our work the specimens cannot be rigidly fixated for clinical reasons (e.g., in a metal skull holder with screw pins), but are held in place with air-pressure cushions, thus the feed-forward rate set to 2 mm/s was decreased by the force-based velocity control to below 1 mm/s on average.

To secure the electrode lead, Leinung and Loth et al. proposed a circular groove of 3 mm length with a diameter of 1.1 ± 0.05 mm with an opening of 0.9 ± 0.05 mm, which provides holding forces equivalent to the technique using a titanium clip on the posterior buttress (18, 19). We hypothesize that the press fit rectangular channel as used in this paper with a similar width (i.e., 1.2 mm channel width) over a much longer length of (i.e., more than 30 mm) on the temporal bone surface combined with the constrained space in the 1.8 mm drill hole to the round window entrance into the cochlea will provide a similar fixation and resulting decrease in electrode migrations. While techniques with small press fit channels have been used, it is yet to be studied if the slight press fit along the longer channel will cause any damage to the electrode.

The electrode is proposed to be placed in a slight press fit into the milled electrode channel, for short-term stability until the healing processes have completed. Studies have shown that a receiver-stimulator in a subperiosteal pocket without milled bone recess will still spontaneously form one after some time through osteoclasts, assumably because of the applied pressure (66–68). It stands to reason that the press fit would also apply pressure on the channel walls, and thus the channel might widen up underneath the surface. At the same time, it has been reported that the implants become encased in fibrotic tissue with intense contact with the underlying bone, holding them in place, and potentially providing long-term stability (6).

The channel depth errors were slightly biased toward shallow channels, with a mean deviation from the planned channel depth of 0.10 mm. However, the errors are greater than what is observed in the depth displacement, where the accuracy in depth was 0.01 mm. Further analysis revealed that the bias of about 0.1 mm stemmed from a difference in temporal bone segmentation, where in most cases the reconstructed surface mesh from the planning CBCT was elevated with respect to the postoperative surface from the micro-CT. Possible explanations are the tendency to over-segment (i.e., include more voxels to the volume) to avoid holes in the planning surface, and the fact that the periosteum was not yet removed at the time when the planning CBCT was taken.

During the milling, the temperatures of the surrounding bone should not exceed 47°C degrees for more than 1 min such as not to cause thermal osteonecrosis (69). This issue is seen as unproblematic in the current work since the thin and shallow channels are created in only a couple of minutes in total, the tool is in constant motion, and the heat is conducted away by the constant irrigation. External irrigation mostly has a major impact by clearing the flutes from bone chips and, if insufficient, cooling the drill between drilling intervals could be introduced, as the heat in the bone is not dissipated quickly due to its low thermal conductivity (70).

The proposed fixation technique is only a viable option in terms of cost if the robotic system is also used for the other steps of cochlear implantation. The proposed technique here does not foresee the robotic milling of an implant bed for the receiver-stimulator, only a ramped bone recess into the channel for the fantail. However, based on the surgeon's preference the robotic milling of the implant bed for the receiver-stimulator could be added. Right now, this would entail a bigger incision and a retraction of the periosteum, such that the robot can access that area with its milling tool close to perpendicular to the bone surface as when milling the channel. Milling at an acute angle to the bone surface, as it is done manually by the surgeon under the lifted skin flap, is difficult with the current five-axis robot arm design. This is because the tool tracking marker would then be oriented toward the patient and would hardly be visible by the tracking camera. Just as the full implant bed could be added, so could smaller recesses for the individual cochlear implant types with pedestals, pins, and screw fixations.

Surgeons have created the electrode lead channel manually in the incised and retracted skin so far during this robotic technique. With the concept presented here, the robot creates the electrode channel in the same space and thus does not need a greater skin incision. Exposed bone surface in-between drill tunnel and the electrode lead exit of the receiver-stimulator is required. If there is insufficient space for the required channel length without exceeding curvatures, or also if there is insufficient bone thickness, then the surgeon can again create the electrode lead channel manually over the whole length, or partially in the affected areas.

In this study, the cochlear implants were the same as used for the manual surgery technique. Due to the more direct path from the implant bed to the cochlea, less electrode lead length is required in the robotic technique and thus, shorter channels with smaller bends would potentially be necessary in the future if specific cochlear implants with shorter electrode leads for the robotic technique were developed.

The sharp edges created through the milling of the rectangular channel could be a potential danger to the electrode. A milling tool with a defined cutting depth of the cylindrical milling cutter and a cone-shaped design above that depth could round off the edges of the channel in passing. An additional flat stop could mechanically prevent excessive milling depth for additional safety. Furthermore, safety monitoring systems have been investigated that could detect contact of the milling cutter with the dura mater through determination of the milling condition based on sensor data (e.g., vibrations from a force-torque sensor, electrical impedance, optics) (52, 71).

In the case where full electrode array insertion into the cochlea cannot be achieved, and the insertion depth difference exceeds the length difference that the widening can accommodate, then the widening can be further enlarged manually by the surgeon, or another widening could be introduced.

The process of virtualizing the structures in the surgical site by recording specific points on either the receiver-stimulator mockup or the tripod attachment for the patient marker could be further simplified and made more precise by developing these structures with in-built reference registration points. At the same time, a specific patient marker attachment with an off-center screw design could leave more space for electrode channels.

While the custom-developed prototype planning software enabled an acceptable plan in all 12 cases, it took several iterations per case to get to the desired result. The chosen channel path varied in its shape depending on the patient anatomy, where the position of the middle ear access hole relative to the four surrounding fiducial screws had the biggest impact. The concept of using three Bézier splines with automatic curvature-optimized shapes, connected by two control points might unnecessarily limit the number of solutions of possible electrode channel paths. Future solutions should focus on allowing all possible electrode channel paths with the correct length and acceptable curvatures. The planning times between 9 and 35 min with this prototype software is mostly due to the prototype nature. A fully developed planning software should enable intraoperative planning in a couple of minutes. Since the step of virtualizing the structures of the surgical site took 3 min on average and the robotic milling execution about 4 min, the proposed electrode lead management approach, with intraoperative planning and execution, could be completed in <15 min.

The technology presented above for creating tight-fitting and precisely placed implant beds, with bone removal accuracies with a root mean square error of 0.1 mm and maximal errors below 0.5 mm, could potentially be applied for further hearing implantation surgeries, for example for direct acoustic cochlear stimulators, active middle ear implants, novel vestibular implants (72), or novel drug delivery devices (73). Further possible applications include the creation of access cavities (e.g., mastoidectomy, labyrinthectomy) to tumors such as vestibular schwannomas either using the middle fossa or the retrosigmoid approach. Moreover, cranial flap resection, facial nerve decompression and several neurosurgical approaches are also potentially foreseeable robotic procedures.

## Conclusion

This study investigated robotic milling on the specific use case of surplus electrode lead management during robotic cochlear implantation in an *ex-vivo* model and verified a proposed approach as safe and effective. The approach follows a concept of a non-intersecting electrode lead channel on the temporal bone surface, intraoperatively planned while taking the virtualized surgical site into account and executed with a high-accuracy robotic system. It is designed to provide a standardized, reproducible way of protecting the electrode lead from external trauma and mechanical fatigue due to micro-movements, and to prevent electrode migrations and iatrogenic intracochlear damage. The method of image-guided robotic bone removal in a compliant headrest presented here with average errors below 0.2 mm and maximal errors below 0.5 mm could be used for a variety of other otologic surgical procedures.

## Data Availability Statement

The raw data supporting the conclusions of this article will be made available by the authors, without undue reservation.

## Ethics Statement

The studies involving human participants were reviewed and approved by KEK Bern, Switzerland, Project-ID 2018-00770. The patients/participants provided their written informed consent to participate in this study.

## Author Contributions

SW and JH created the study design. JH and FM developed the hardware components. JH developed the software components. JH, DS, FM, and GO'T carried out the *ex-vivo* experiments. JH analyzed the collected data and wrote the manuscript. All authors reviewed the manuscript and approved the submitted version.

## Funding

This work was supported by the Swiss National Science Foundation SNF (Project 176007). Blackberry QNX provided an Academic Key for the QNX Software Development Platform. Funding in the form of cochlear implantation surgery equipment (e.g., Mi1250 Implant Template Ms040107, Mi1250 Silicone Mock-up Implants) was provided by MED-EL.

## Conflict of Interest

SW is cofounder, shareholder, and chief executive officer of CASCINATION AG (Bern, Switzerland), which commercializes the robotic cochlear implantation technology. The remaining authors declare that the research was conducted in the absence of any commercial or financial relationships that could be construed as a potential conflict of interest.

## Publisher's Note

All claims expressed in this article are solely those of the authors and do not necessarily represent those of their affiliated organizations, or those of the publisher, the editors and the reviewers. Any product that may be evaluated in this article, or claim that may be made by its manufacturer, is not guaranteed or endorsed by the publisher.
