# Supplementary figures and images for: Robotic Milling of Electrode Lead Channels During Cochlear Implantation in an ex-vivo Model
Source: Front Surg. 2021 Nov 11;8:742147. doi: 10.3389/fsurg.2021.742147 (PMC8631814; doi:10.3389/fsurg.2021.742147)

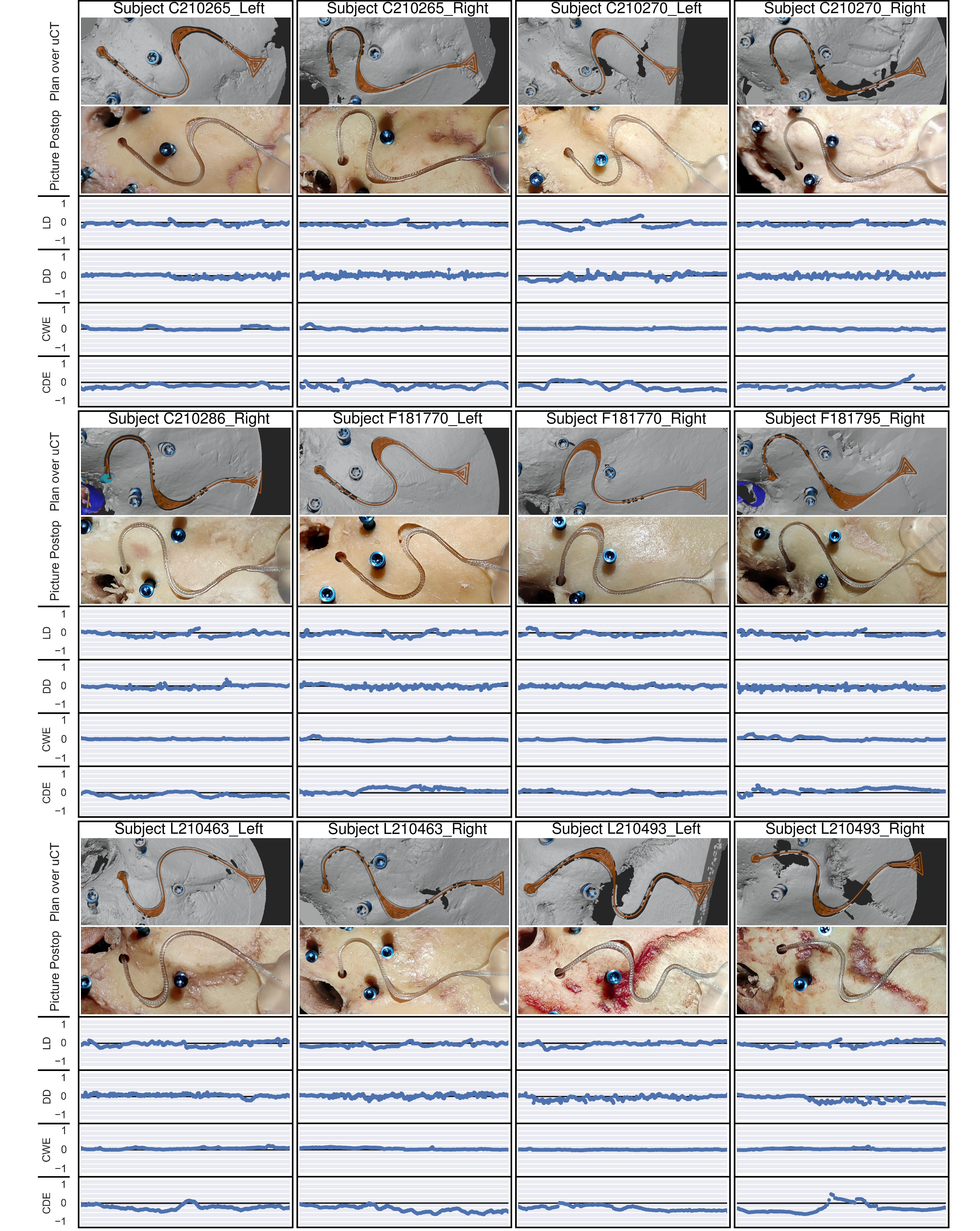

Supplement: Supplementary file 2 [file Image_1.JPEG]
